# Prognostic values of high sensitivity cardiac troponin T and I for long-term mortality in hemodialysis patients

**DOI:** 10.1038/s41598-022-17799-4

**Published:** 2022-08-17

**Authors:** Kajohnsak Noppakun, Kannika Ratnachina, Nichanan Osataphan, Arintaya Phrommintikul, Wanwarang Wongcharoen

**Affiliations:** 1 Division of Nephrology, Department of Internal Medicine, Faculty of Medicine, Chiang Mai University, Chiang Mai, 50200 Thailand; 2 Division of Cardiology, Department of Internal Medicine, Faculty of Medicine, Chiang Mai University, Chiang Mai, 50200 Thailand; 3 Center for Medical Excellence, Faculty of Medicine, Chiang Mai University, Chiang Mai, 50200 Thailand; 4 Pharmacoepidemiology and Statistics Research Center (PESRC), Faculty of Pharmacy, Chiang Mai University, Chiang Mai, 50200 Thailand

**Keywords:** Cardiology, Nephrology

## Abstract

Previous studies using contemporary cardiac troponin (cTn) assays have shown conflicting results in predictability of mortality and major adverse cardiovascular events (MACEs) in hemodialysis patients. We aimed to evaluate the prognostic values of high-sensitivity cTnT (hs-cTnT) and hs-cTnI for long-term mortality and MACEs in asymptomatic chronic hemodialysis patients. 198 asymptomatic patients undergoing regular hemodialysis (age 62.4 ± 14.8 years) were enrolled. Pre-dialysis hs-cTnT and hs-cTnI levels were measured. The study outcomes were long-term all-cause mortality and MACEs. Median values of hs-cTnT and hs-cTnI were 61.1 ng/L (IQR 36.6–102.0) and 18.4 ng/L (IQR 9.5–36.6), respectively. During a median follow-up of 13.5 months, 30 (15.1%) patients developed MACEs, and 20 (10.1%) patients died. The patients in highest quartile of hs-cTnT level (≥ 102 ng/L) had increased risk of long-term mortality (HR 3.34; 95%CI 1.39–8.04, P = 0.005). However, hs-cTnI levels above highest quartile (≥ 36 ng/L) was not significantly associated with increased risk of all-cause mortality. Nevertheless, elevated level of hs-cTnT and hs-cTnI was associated with increased risk of MACEs. We demonstrated that higher level of hs-cTnT, but not hs-cTnI, was associated with increased risk of long-term mortality. Nevertheless, higher level of hs-cTnT and hs-cTnI both were associated with greater risk of long-term MACEs.

## Introduction

It is well-established that the patients with chronic hemodialysis had a markedly higher rate of cardiovascular death and all-cause death than the general population^1^. Early recognition of patients undergoing hemodialysis with heightened mortality risk is essential for the early intervention and attentive therapy. Multiple biomarkers have been explored to be used as a predictor of long-term adverse outcome in this population^2^. Several investigators have shown that asymptomatic patients with end-stage renal disease (ESRD) have chronically elevated levels of cardiac troponin (cTn)^2–6^. Recent studies have demonstrated that nearly all of patients with regular dialysis had a raised level of high-senstivity cTnT (hs-cTnT), while around one-third of patients had an increased level of high sensitivity cTnI (hs-cTnI)^6–9^. The dissimilar findings between cTnT and cTnI may be attributable to the distinctions in cellular distribution, biochemical, genetic, and kinetic features^10,11^. With this regard, it has been suggested that hs-cTnI may be more favorable in the diagnostic use of patients with suspected acute coronary syndrome^6^. However, in terms of prognostic values between cTnT and cTnI in patients with ESRD, the data are still conflicting^7,12,13^. In addition, conventional cardiac troponin assay used in those studies may have drawback of limited sensitivity and specificity as compared to high-sensitivity assay. Based on the revised definition of myocardial infarction with strict recommendations on test precision, the high sensitivity assay of cardiac troponin T and I has gained popularity in its use. As a result, we aim to evaluate and compare the prognostic value of the hs-cTnT and hs-cTnI assay for long-term mortality and major adverse cardiovascular events (MACEs) in asymptomatic patients receiving regular hemodialysis.

## Methods

### Study population

In this prospective observational cohort study, ESRD patients, aged more than 18 years, who had undergone regular hemodialysis for more than 3 months at the hemodialysis unit at Chiang Mai University hospital were enrolled. All of the participants who had an acute myocardial infarction, heart failure, or pulmonary embolism within the past 6 months; recent major or traumatic surgery within the last 4 weeks; and recent coronary and/or valvular surgery within the last 6 months were excluded from this study. A total of 200 patients with chronic hemodialysis were screened for eligibility. Two patients were excluded due to the occurrence of acute coronary syndrome in one patient and heart failure requiring hospitalization in one patient within the past 6 months. As a result, there were 198 asymptomatic patients with chronic hemodialysis enrolled in this study during a period of September 2020 to January 2021. Patients who underwent a kidney transplant or who were lost to follow-up were censored on the day of the transplant as well as the day of their final visit. This study was approved by the institutional research board of Faculty of Medicine, Chiang Mai University (Approval No. 182/2564). Informed consent and all procedure followed the Declaration of Helsinki and ethical standards of the responsible committee on human experimentation.

### Clinical data and laboratory measurements

Clinical data were acquired at the time of study entry, including age, gender, cardiovascular risk factors, medication, comorbidity, blood chemistry, and hemodialysis data (dialysis duration, frequency, and access). The pre-dialysis hs-cTnT and hs-cTnI levels were measured in all subjects (single measurement). Several studies have revealed that cardiovascular events are more frequently after a long interdialytic interval than after a short interdialytic interval during hemodialysis sessions^14,15^. Therefore, we chose to obtain the blood sample of pre-dialysis hs-cTnT and hs-cTnI levels during the long dialytic period. The hs-cTnT was analyzed by electroluminescence immunoassay using Cobas e801 (Roche Diagnostics) and hs-cTnI with the ARCHITECT i2000SR Diagnostic System (Abbott). The limit of detection was 3 ng/L for hs-cTnT and 3.2 ng/L for hs-cTnI, and the coefficients of variation below 10% were 13 ng/L and 4.7 ng/L for hs-cTnT and hs-cTnI. The cutoff at the 99th percentile was 14 ng/L for hs-cTnT and 26.2 ng/L for hs-cTnI.

### The study endpoints

All participants were followed for the first occurrence of all-cause mortality and MACEs until September 2021. The primary endpoint was the prognostic values of hs-cTnT and hs-cTnI on all-cause mortality, with the secondary endpoint being the prognosis for MACEs. MACEs were defined as a composite outcome of nonfatal myocardial infarction, heart failure hospitalization, and death from any cause.

### Statistical analysis

Continuous variables were expressed as means and standard deviations when normally distributed, or medians and interquartile ranges when not normally distributed. The comparison across the different groups was performed using the Mann–Whitney U test or the Student t-test. Categorical variables were presented as frequency (%) and compared between groups using Fisher's exact test. A Kaplan–Meier time-to-event curve with a log-rank test was performed to compare the outcome by cardiac troponin quartile. The Cox proportional hazards regression model was used to assess the association between cardiac troponin levels and the risk of adverse cardiovascular events. The prognostic value of hs-cTnT and hs-cTnI on all-cause mortality and MACEs was determined as a hazard ratio (HR) and 95% confidence interval (CI). Statistical significance was defined as a P-value of less than 0.05. The statistical software package SPSS version 23 (IBM Corp., Armonk, NY, USA, https://www.ibm.com/products/spss-statistics) was used for statistical analysis and STATA version 16.2 (StataCorp, College Station, TX, USA, https://www.stata.com/order/new/edu/profplus/campus-profplus/) was used for graphic creation.

### Ethics approval and consent to participate

The difference in prognostic value between high‑sensitive cardiac troponin T and I for long-term major adverse cardiovascular events in hemodialysis patients was approved by the ethics committee of the Faculty of Medicine, Chiang Mai University, approval number 182/2564. The investigations were carried out in accordance with the Declaration of Helsinki, including written informed consent of all participants.

## Results

### Study population and outcomes

A total of 198 asymptomatic patients with chronic hemodialysis were enrolled in this study. The median follow-up durations were 13.5 months, one patient lost to follow up and one received a kidney transplant. Table 1 shows baseline clinical characteristics and demographic data. The mean age of the patients was 62.4 ± 14.8 and 111 (56.1%) were male. Our studied population had a high prevalence of cardiovascular risk factors. There were 180 (90.9%) patients with hypertension, 89 (44.9%) patients with diabetes and 105 (53%) patients with dyslipidemia. The history of coronary artery disease, atrial fibrillation and cerebrovascular disease was evident in 26 (13.1%) patients, 23 (11.7%) patients, and 21 (10.6%) patients. The median dialysis vintage was 37.2 months. There were 187 (94.4%) patients receiving hemodialysis three times a week and the 11 (5.6%) receiving hemodialysis twice a week. The median hs-cTnT level was 61.1 (36.6–102.0) ng/L, with 196 (99%) individuals having hs-cTnT levels higher than the cutoff point (99th percentile upper reference limit of 14 ng/L). The median hs-cTnI level was 18.4 (9.5–36.6) ng/L, with hs-cTnI levels above the cutoff point (99th percentile upper reference limit of 26.2 ng/L) present in 68 (34.3%) participants. It has been demonstrated that that the approved 99th-percentile of hs-cTnI (26.2 ng/L) may not be biologically equivalent of the 99th-percentile of hs-cTnT (14 ng/L). With this regard, we also used the cutoff point of 8.7 ng/L in female and 16 ng/L in male for the hs-cTnI^16,17^. There were 127 (64.1%) patients who had level of hs-cTnI above this gender-specific cutoff point.

**Table 1:** Baseline characteristics of the population.

Characteristics	Total (N = 198)	Alive (N = 178)	Dead (N = 20)	P-value
Mean age, years	62.4 ± 14.8	61.3 ± 14.7	72.8 ± 11.8	< 0.001
**Gender**				1.000
Male	111 (56.1%)	100 (56.2%)	11 (55.0%)	
Female	87 (43.9%)	78 (43.9%)	9 (45.0%)	
Mean body mass index (kg/m^2^)	23.1 ± 4.9	23.3 ± 5.2	21.4 ± 2.6	0.108
**Smoking status**				0.695
No smoking	144 (72.7%)	130 (73.0%)	14 (72.7%)	
Ex-smoker	41 (20.7%)	35 (19.7%)	6 (30.0%)	
Current smoker	4 (2%)	4 (2.2%)	0 (0%)	
**Comorbidities**				
Diabetes mellitus	89 (44.9%)	79 (44.4%)	10 (50.0%)	0.681
Dyslipidemia	105 (53.0%)	94 (52.8%)	11 (55.0%)	1.000
Hypertension	180 (90.9%)	161 (90.9%)	19 (95.0%)	1.000
Atrial fibrillation	23 (11.7%)	18 (10.2%)	5 (25.0%)	0.065
Coronary artery disease	26 (13.1%)	23 (12.9%)	3 (15.0%)	0.733
Cerebrovascular disease	21 (10.6%)	19 (10.7%)	2 (10.0%)	1.000
Chronic obstructive pulmonary disease	2 (1%)	1 (0.6%)	1 (5.0%)	0.193
**Medication**				
Antiplatelet	71 (35.9%)	64 (36.2%)	7 (35.0%)	1.000
Beta-blockers	126 (63.6%)	113 (66.1%)	13 (65.0%)	1.000
ACEi/ARB	51 (25.8%)	41 (23.9%)	10 (50.0%)	0.029
Calcium channel blockers	127 (64.1%)	114 (66.7%)	13 (65.0%)	1.000
Statins	112 (56.6%)	102 (59.6%)	10 (50.0%)	0.475
Oral anticoagulant	18 (9.1%)	12 (7.0%)	6 (30.0%)	0.005
**Dialytic data**				
Dialysis vintage (months), median (IQR)	37.2 (22.4–60.3)	37.5 (22.9–60.2)	34.8 (21.9–61.2)	0.773
**Dialysis frequency**				1.000
2 times per week	11 (5.6%)	10 (5.6%)	1 (5.0%)	
3 times per week	187 (94.4%)	168 (94.4%)	19 (95.0%)	
**Dialysis access**				0.619
Arteriovenous fistula	138 (69.7%)	126 (70.8%)	12 (63.2%)	
Arteriovenous graft	8 (4%)	7 (3.9%)	1 (5.3%)	
Perm catheter	50 (25.4%)	44 (24.7%)	6 (31.6%)	
Double lumen catheter	1 (0.6%)	0 (0.0%)	1 (0.5%)	
**Laboratory data**				
Serum sodium (mEq/L)	136.7 ± 3.3	136.8 ± 3.3	135.9 ± 3.4	0.210
Serum potassium (mEq/L)	4.4 ± 0.6	4.5 ± 0.6	4.4 ± 0.6	0.526
Hemoglobin (g/dL)	10.4 ± 1.4	10.5 ± 1.4	9.6 ± 1.1	0.027
Serum albumin (g/dL)	4.0 ± 0.4	4.0 ± 0.4	3.5 ± 0.5	< 0.001
hs-cTnI (ng/L), median (IQR)	18.4 (9.5–36.6)	17.2 (9.4–33.4)	27.3 (13.3–43.4)	0.105
≥ 26.2 ng/L	68 (34.3%)	58 (32.6%)	10 (50%)	0.139
hs-cTnT (ng/L), median (IQR)	61.1 (36.6–102.0)	59.0 (36.0–95.8)	111.4 (56.4–174.1)	0.002
≥ 14 ng/L	196 (99%)	176 (98.9%)	20 (100.0%)	1.000

Among 198 patients with regular dialysis, the first occurrence of MACEs was reported in 30 patients (15.2%), including nonfatal myocardial infarction in 7 patients, heart failure hospitalization in 7 patients, and death from any cause in 16 patients. Among 30 patients who developed MACEs, 2 patients with nonfatal myocardial infarction and 2 patients with heart failure hospitalization subsequently died in separate events. As a result, a total of 20 (10.1%) patients died during follow-up, in which cardiovascular death occurred in 9 patients (4.5%).

The non-survivors were significantly older than the survivors (72.8 ± 11.8 vs. 61.3 ± 14.7 years, p < 0.001). Atrial fibrillation was non-significantly more prevalent in non-survivors (25.0% vs. 10.2%, P = 0.065), leading to the greater use of oral anticoagulants in non-survivors compared to survivors (30.0% vs. 7.0%, P = 0.005). The higher use of angiotensin converting enzyme inhibitor (ACEI) or angiotensin receptor blocker (ARB) was also evident in non-survivor group (23.9% vs. 5.0%, P = 0.029). The presence of other co-morbidities did not differ between non-survivors and survivors. The significantly lower levels of hemoglobin and serum albumin were observed in non-survivors. Interestingly, non-survivors had significantly greater hs-cTnT levels compared to survivors (111.4, IQR 56.4–174.1 vs. 59.0, IQR 36.0–95.8, P = 0.002), while hs-cTnI levels were not statistically different between two groups (p = 0.105) (Table 1).

### Prognostic values of hs-cTnT and hs-cTnI on long-term outcomes

We classified hs-cTnT and hs-cTnI into quartiles based on concentration. Regarding the hs-cTnT level, the first quartile, the second quartile, the third quartile and the fourth quartile had hs-cTnT levels < 36.5 ng/L, 36.5–61.0 ng/L, 61.1–101.9 ng/L, and ≥ 102.0 ng/L, respectively. With respect to the hs-cTnI level, the first quartile, the second quartile, the third quartile and the fourth quartile had hs-cTnI levels < 9.5 ng/L, 9.5–18.3 ng/L, 18.4–35.9 ng/L, ≥ 36.0 ng/L. To analyze the association between hs-cTn and clinical outcomes, hs-cTn data were compared between patients with values above the highest quartile of hs-cTnT (≥ 102 ng/L) and hs-cTnI (≥ 36 ng/L) and those with values below the highest quartile. The univariable and multivariable Cox regression analysis of hs-cTnT and hs-cTnI on long-term all-cause mortality were shown in Table 2. When compared to the lower quartile, patients in the highest quartile of hs-cTnT levels showed a higher risk of all-cause mortality (unadjusted HR 3.34; 95%CI 1.39–8.04, P = 0.005). The relationship between hs-cTnT and all-cause mortality remained significant after adjusting for age and gender (adjusted HR 2.90; 95%CI 1.20–7.03, P = 0.018). On the other hand, hs-cTnI levels above the highest quartile was not significantly associated with increased risk of all-cause mortality (unadjusted HR 2.10; 95%CI 0.86–5.13, P = 0.105 and adjusted HR 1.94; 95%CI 0.79–4.78, P = 0.147). To prevent the overfitting in the multivariate regression model, the number of potential variables adjusted in the Cox regression model for all-cause mortality was limited. As a result, only age and gender were adjusted in the main analysis. Nevertheless, we performed exploratory analysis to confirm the validity of multiple regression analysis. All variables with significant differences between the survivors and non-survivors were also adjusted in the Cox regression model. We demonstrated that the hs-cTnT (adjusted HR 2.64 [1.02–6.78]), but not hs-TnI (adjusted HR 0.95 [0.31–2.85]), remained significantly associated with an increased risk of all-cause mortality after adjusting for age, atrial fibrillation, serum albumin, hemoglobin, the use of ACEi/ARB and the use of oral anticoagulant.

**Table 2:** Univariable and multivariable analysis of hs-cTnT and hs-cTnI on primary and secondary outcomes.

Variables	All-cause mortality		MACEs	
	HR (95%CI)	P-value	HR (95%CI)	P-value
**hs-cTnT above highest quartile (≥ 102 ng/L)**				
Unadjusted OR	3.34 (1.39–8.04)	0.007	2.57 (1.25–5.30)	0.010
Adjusted OR^a^	2.90 (1.20–7.03)	0.018	2.27 (1.09–4.71)	0.028
**hs-cTnI above highest quartile (≥ 36 ng/L)**				
Unadjusted OR	2.10 (0.86–5.13)	0.105	2.51 (1.22–5.19)	0.012
Adjusted OR^b^	1.94 (0.79–4.78)	0.147	2.25 (1.07–3.35)	0.032

^a^Adjusted for age and gender.

^b^Adjusted for age, gender, and atrial fibrillation.

A cut-off level of hs-cTnT ≥ 102 ng/L provided a sensitivity of 50%, a specificity of 78.1%, a positive predictive value of 20.4% and a negative predictive value of 93.0% to predict long-term mortality. Apart from the level of hs-cTnT, age was also another independent predictor of long-term mortality.

**Figure 1:**
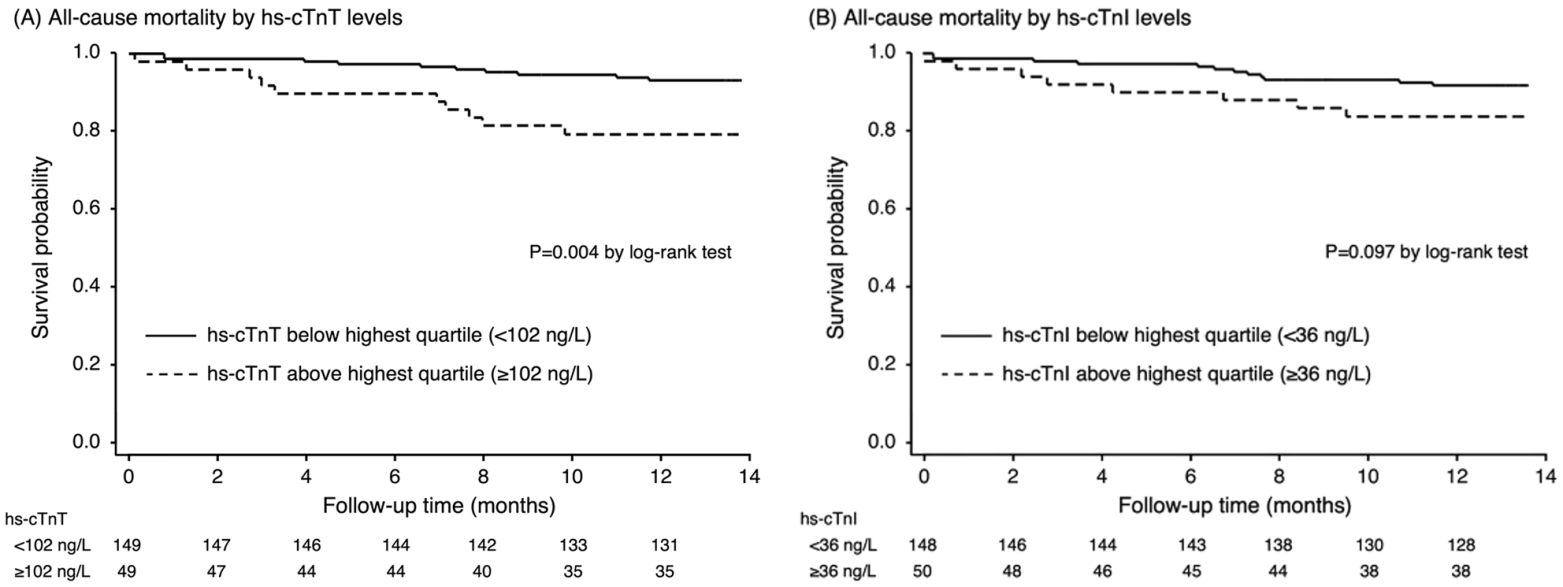
Kaplan–Meier survival curve analysis for all-cause mortality in patients above or below the highest quartile of hs-cTnT (**A**) and hs-cTnI (**B**).

**Figure 2:**
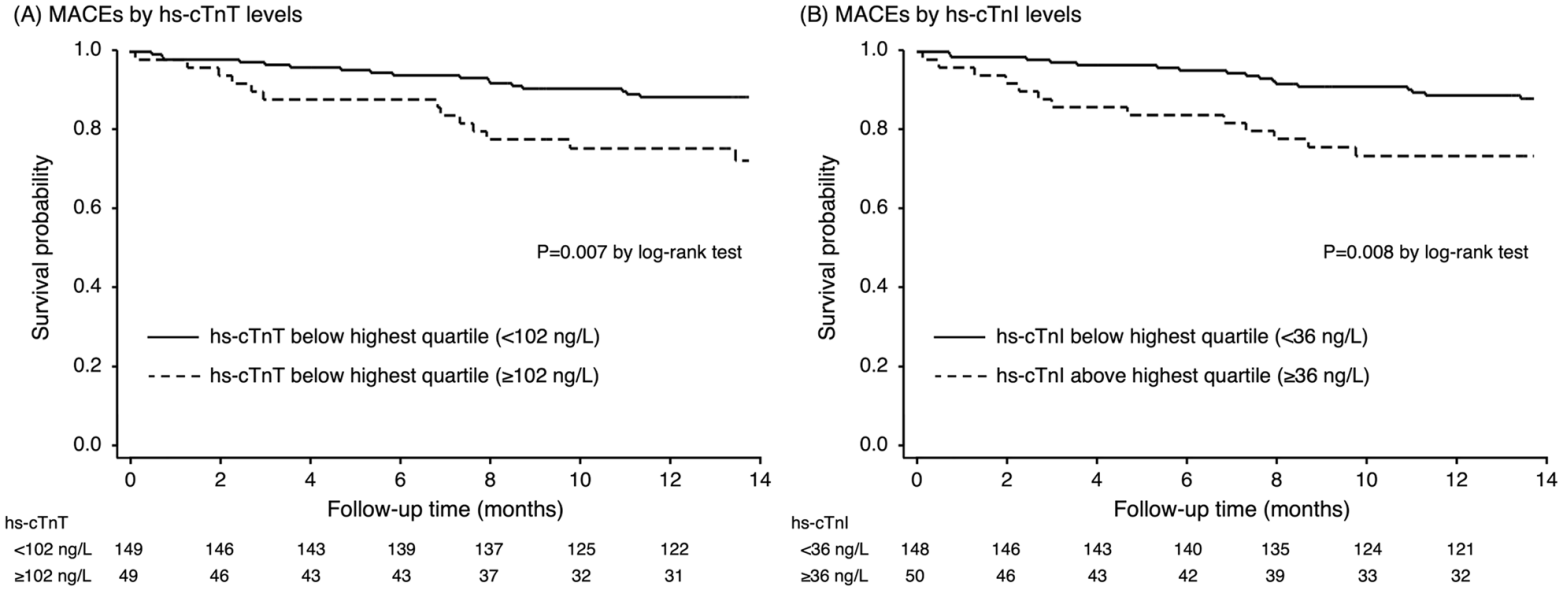
Kaplan–Meier survival curve analysis for MACEs in patients above or below the highest quartile of hs-cTnT (**A**) and hs-cTnI (**B**).

According to the secondary outcome, patients with hs-cTnT level in the highest quartile was associated with a greater risk of MACEs (HR 2.57; 95%CI 1.25–5.30, P = 0.010) compared to those in the lower quartile. (Table 2) After adjusting for age, gender, and atrial fibrillation, the hs-cTnT remained significantly associated with the increased risk of MACEs (HR 2.27; 95%CI 1.09–4.71, P = 0.028). Similar to hs-cTnT level, hs-cTnI level demonstrated a significant relationship with the occurrence of MACEs (unadjusted HR 2.51; 95%CI 1.22–5.19, P = 0.012 and adjusted HR 2.25; 95%CI 1.07–3.35, P = 0.032). Figures 1 and 2 shows the Kaplan–Meier curves of the primary and secondary outcomes.

## Discussion

We demonstrated that nearly all of patients with chronic hemodialysis had an increased level of hs-cTnT and one-third of patients had an elevated level of hs-cTnI, similar to previous studies^6,18,19^. Besides coronary artery disease, other factors enhancing the level of hs-cTn in hemodialysis patients include left ventricular hypertrophy, impaired left ventricular systolic and diastolic function, volume overload, and repeatedly intradialytic hypotension^18,20^. These conditions can lead to myocardial injury and myocardial necrosis. Consequently, the patients with these circumstances were more likely to develop cardiovascular adverse events. With this regard, the predictability of hs-cTn on long-term cardiovascular events and mortality has been explored in several studies. As hs-cTnT differs from hs-cTnI in several aspects including the cellular distribution, biochemical profiles and analytical performances^10,11^, it is possible that these two biomarkers may have different prognostic values on long-term outcome.

Our study showed that hs-cTnT and age were two independent predictors of long-term mortality in patients receiving regular hemodialysis. In contrast, hs-cTnI level was not significantly related with long-term mortality. Nevertheless, higher levels of hs-cTnT and hs-cTnI both were associated with an increased risk of long-term MACEs. Our findings were in line with previous studies. One used contemporary assay^7^ and the others used high-sensitivity assays in their studies^3,8^. They showed that cTnT but not cTnI predicted long-term adverse outcomes.

The reason why hs-cTnT had a greater prognostic value on long-term mortality is unclear. Previous study has demonstrated a higher tissue concentration and free cytoplasmic concentrations in cTnT compared to cTnI. In addition, cTnT was observed in blood of patients with acute myocardial infarction as a mixture of complexed (cTnT-I-C) and free cTnT. On the contrary, cTnI was more hydrophobic and was detected dominantly in the binary complex (I-C) form, with lesser extents of the ternary (T-I-C) complex. Degradation of the complex led to more rapidly clearance of the protein^11,21^. With this regard, we proposed that hs-cTnT was possibly more sensitive to myocardial injury than hs-cTnI due to the higher concentration in tissue and free cytoplasm. Moreover, hs-cTnT may release more early with greater amount than hs-cTnI during myocardial injury. This disparity feasibly account for the much higher prevalence of elevated level of hs-cTnT in asymptomatic patients receiving regular hemodialysis, compared to hs-cTnI level. In addition, previous studies from our group and from Buiten et al. demonstrated similar findings that hs-cTnT was superior to hs-cTnI in predicting patients with history of coronary artery disease^6,22^. Therefore, patients with higher level of hs-cTnT may have higher prevalence of subclinical coronary artery disease which may contribute to the increased risk of long-term mortality.

Nevertheless, some studies have reported the significant association of hs-cTnI and long-term adverse outcomes. Gaiki and colleagues determined the prognostic value of hs-cTnI in asymptomatic patients with hemodialysis. At a 2-year follow-up, they discovered that using a hs-cTnI was predictive of cardiac events^23^. The Vitros ES assay manufactured by Ortho Clinical Diagnostics was used in that study. Other studies from Artunc and colleagues have also shown that hs-cTnI was associated with long-term mortality. The troponin I Ultra assay on a Siemens ADVIA Centaur system was used in their studies^18,24^. On the other hands, our study along with others which demonstrated the lack of predictive power of hs-cTnI and adverse outcomes used hs-cTnI assay manufactured by Abbott Diagnostics^3,8^. With this regard, the diversity of predictive power of hs-cTnI among studies may be resulted from the use of different hs-cTnI assay.

Our study confirmed that elevated level of hs-cTnT can be used as a prognostic marker for long-term mortality in patients with regular hemodialysis. The optimal cutoff level of hs-cTnT for predicting the long-term mortality is unknown. We proposed the cutoff hs-cTnT level of 102 ng/L to predict long-term mortality with a sensitivity of 50%, a specificity of 78.1%, a positive predictive value of 20.4% and a negative predictive value of 93.0%.

Our study has a number of limitations. First, our study cohort was recruited from a single dialysis center and our sample size was relatively small. As a result, the generalizability of our findings is limited. Second, we did not include patients with chronic peritoneal dialysis. Therefore, our findings could not be applied in these populations. Third, since cardiac troponin levels were measured only once, it was difficult to show how changes in biomarkers affected the clinical outcome of patients. Fourth, other biomarkers that may have predicted the outcome such as C-reactive protein were not assessed in our studied population. Fifth, the constant effect of a predictor on the outcomes is crucial. Previous study from our group showed that the levels of pre-dialysis hs-cTnI and T did not differ between short interdialytic and long interdialytic intervals^6^. Another small study demonstrated that the majority of patients with asymptomatic hemodialysis had relatively constant troponin values after 3 consecutive monthly measurements. Nevertheless, 20% of them had fluctuating troponin values over a 3-month period^25^. Whether the serial measurement of hs-cTn is a better predictor on long-term outcome than single measurement merits further studies.

## Conclusions

We demonstrated that elevated hs-cTnT level, but not hs-cTnI, was associated with a higher risk of long-term mortality. Nevertheless, higher level of hs-cTnT and hs-cTnI both were associated with greater risk of long-term MACEs in patients with chronic hemodialysis.

## Data Availability

The difference in prognostic value between high‑sensitive cardiac troponin T and I for long-term major adverse cardiovascular events in hemodialysis patients does not cover data posting in public databases. However, data are available upon request should be sent to bwanwarang@yahoo.com and are subject to approval by the Faculty of Medicine, Chiang Mai University Ethics Committee.
